# Development of a Global Subjective Skin Aging Assessment score from the perspective of dermatologists

**DOI:** 10.1186/s13104-019-4404-z

**Published:** 2019-06-28

**Authors:** Punnapath Buranasirin, Krit Pongpirul, Jitlada Meephansan

**Affiliations:** 1 Division of Dermatology, Department of Medicine, Chulabhorn International College of Medicine, Thammasat University, Pathum Thani, Thailand; 2 Department of Preventive and Social Medicine, Faculty of Medicine, Chulalongkorn University, Bangkok, 10330 Thailand; 3 Department of International Health, Johns Hopkins Bloomberg School of Public Health, Baltimore, MD USA

**Keywords:** Skin aging assessment, Dermatology, Attitude

## Abstract

**Objective:**

Several skin aging assessment scales exist but no standard scale is widely used. Dermatologists may be the most appropriate persons for skin assessment but their perceptions regarding the signs of skin aging are unexplored. To develop a simple global skin aging assessment score from the perspective of dermatologists, an online questionnaire, the Thai Dermatologist Survey of Skin Aging Assessment, was conducted from October to December, 2016. Twenty-nine signs with published evidence of their relevancy to skin aging were included. Certified dermatologists were asked to score each sign using a 5-point Likert scale. Descriptive statistics and exploratory factor analysis (EFA) were used for data analysis.

**Results:**

Of 213 randomly selected dermatologists, 145 responded to the survey. EFA revealed 3 important factors related to skin aging: Factor 1 comprised 8 signs related to atrophy (deep/superficial wrinkles, eye bags, lax appearance, etc.); Factor 2 comprised 7 signs related to discoloration (freckles, lentigines, melasma, etc.); and Factor 3 comprised 3 malignant skin lesions. The Global Subjective Skin Aging Assessment (GS2A2) score is a simple numerical score that can be used to evaluate the anti-aging effects of a cosmetic product or dermatologic intervention. This score should be tested further for validity and reliability.

**Electronic supplementary material:**

The online version of this article (10.1186/s13104-019-4404-z) contains supplementary material, which is available to authorized users.

## Introduction

Nowadays, the aging society is increasing and concerns about aging sequence have started to receive special attention. The primary target of an aging study is to increase the quality of elderly life and to prevent age-related diseases. Especially, aged skin is more interesting because the skin is the most noticeable sign of the aging mechanism and demonstrative of human health, as it seems to prognosticate the systemic illness and prognosis.

Chronologic age and solar damage are the most important intrinsic and extrinsic factors associated with skin aging. Skin aging characteristics vary ethnically among races. No one is spared from skin wrinkling, sunspots, uneven skin color, vascular abnormality, benign tumor, and skin cancer; yet, these symptoms manifest differently according to one’s ethnic origin. Among the races, Caucasians manifest signs of photo-damage earlier than other races because of their relatively low melanin content [1]. Furthermore, Caucasians manifest earlier onset of skin aging together with more obvious skin wrinkles and sagging signs than other races [2]. Additionally, the clinical manifestation of skin aging among Asians differs considerably compared to Caucasians. In Asians, pigmentation changes are the most frequent and significant manifestation of skin aging [3]. Despite this fact, the mechanisms responsible for the deleterious effects and cutaneous alterations are distinct, and general skin signs are essential for the clinical assessment of skin aging. An ideal skin aging assessment should include all signs of skin aging.

As features of skin aging depend on genetics, race, age, sex, and exposure to different external factors [4], developing a global skin aging assessment scale has been difficult. Most skin aging scales have been used for research studies [5]. Currently, there are more than 100 skin aging assessment scales. They have been proposed and used in clinical practice and in research to compare treatment outcomes. Some assessment methods have used only selected signs, such as those from the Glogau [6] and Fitzpatrick classification systems [7], focused on specific areas (e.g., the face, neck, and chest) [8] or a specific ethnicity [9]. Others have applied multidimensional assessment of multiple skin signs but the content and measurement properties were sub-standard [10].

From 1978 to 2016, only 5 published multidimensional assessment scales were determined to have high methodological quality for all skin types according to the COnsensus-based Standards for the selection of health Measurement INstruments (COSMIN)—a group that developed a critical appraisal tool (checklist) containing the standards for evaluating the methodological quality of studies investigating the properties of health measurement instruments [10]. Of these, 5 scales are known as the Merz Aesthetics Scale (MAS) [11], SCore of INtrinsic and EXtrinsic skin Aging (SCINEXA) [12], Skin Aging Score (SAS) [13], and two unnamed scales (Table 1) [14, 15]. Additionally, there are some limitations: most scores focus on Caucasians, they do not have complete skin aging signs, they are not widely used, and they do not have clear terminology. All of these scales were developed based on the relationship between skin aging signs and many factors that involve aging. Dermatologists are important persons who diagnose any skin signs because of their expertise and experience. However, none of these scales were designed to perform an assessment from a dermatologist’s perspective.

**Table 1:** Evaluation of five multidimensional scores using COSMIN framework

Scale name	Author	Measurement property
SAS	Guinot et al. [13]	Excellent structural validity Excellent criterion validity
SCINEXA	Vierkotter et al. [12]	Good hypothesis testing Good criterion validity
MAS	Rzany et al. [11]	Good reliability Good hypothesis testing Good criterion validity
N/A	Allerhand et al. [14]	Excellent reliability Excellent structural validity
N/A	Bazin and Flament [15]	Excellent reliability Excellent structural validity

*COSMIN* COnsensus-based Standards for the selection of health Measurement INstruments, *SAS* Skin Aging Score, *SCINEXA* SCore of INtrinsic and EXtrinsic skin Aging, *MAS* Merz Aesthetics Scale, *N/A* not applicable

Today, there are many techniques and instruments to detect skin aging for research and treatment, including the 3-dimensional camera [16], dermoscopy [17], a physical sample analysis of color [18], and measurement of elasticity [19]. However, these include only some of the signs of skin aging. Some cosmetic products can improve skin elasticity, but changes in other clinical symptoms may be unclear. In addition, pigmentation and malignancy may require a dermatologist to identify signs of aging.

This is the first study that aimed to develop a simple global skin aging assessment score based on the perceptions of certified dermatologists.

## Main text

### Materials and methods

#### Design and participants

This study was conducted between October 1 and December 31, 2016. An internet-based survey using Google form was delivered to randomly selected dermatologists certified by the Dermatology Society of Thailand, including those with board certification, Doctor of Philosophy in Dermatology, Master of Science in Dermatology, and Fellowship and Diploma in Dermatology.

#### Questionnaire

The questionnaire comprised three main sections. The first section asked about respondents’ demographic characteristics and clinical training and experience with no identifiable data. In the second section, all skin signs of the five COSMIN-qualified checklists were reviewed. To ensure that all possible and commonly known signs were included, two major textbooks [20, 21] that have been widely used for dermatology training in Thailand were also reviewed. To ensure scale simplicity, skin signs that required facial expression or were localized to specific facial areas were excluded. Therefore, 29 signs with published evidence of their relevancy to skin aging were included (Table 2). Respondents were asked whether each skin sign was essential for a skin aging diagnosis using the question, “I think that each of the following signs or diseases is essential for skin aging diagnosis” Their responses were based on a 5-point Likert scale (1, strongly disagree; 2, disagree; 3, neither agree nor disagree; 4, agree; 5, strongly agree) (Additional file 1). The third section included the question, “I am familiar with the following scale” to evaluate the familiarity and popularity of currently available, well-developed skin aging assessment scales among Thai dermatologists.

**Table 2:** List of 29 signs and relevant sources

Skin aging signs	Skin aging scales					
	SAS	SCINEXA	MAS	N/A	N/A	Textbooks
Authors:	Guinot et al.	Vierkotter et al.	Rzany et al.	Bazin et al.	Allehand et al.	Lim et al. Yaar et al.
Wrinkles-superficial	Ο	Ο	Ο	Ο	Ο	Ο
Wrinkles-deep	Ο	Ο	Ο	Ο	Ο	Ο
Wrinkles-criscross						Ο
Reduce fat tissue		Ο	Ο			Ο
Nasolabial folds	Ο	Ο	Ο	Ο	Ο	Ο
Eye bags	Ο			Ο	Ο	Ο
Ptosis of eyelids	Ο			Ο	Ο	Ο
Yellowish discoloration		Ο				Ο
Lax appearance/tissue slacking	Ο	Ο			Ο	Ο
Solar elastosis		Ο		Ο		Ο
Pseudoscar		Ο				Ο
Cutis rhomboidalis nuchae		Ο				Ο
Freckles		Ο				Ο
Solar lentigines		Ο				Ο
Melasma		Ο				Ο
Uneven pigment/pigment spot		Ο		Ο	Ο	Ο
Guttate hypomelanosis						Ο
Venous lakes						Ο
Senile purpura						Ο
Telangiectasias		Ο				Ο
Milia	Ο	Ο			Ο	Ο
Sebaceous hyperplasia						Ο
Senile comedone	Ο					Ο
Favre-Racouchot syndrome		Ο				Ο
Actinic keratosis		Ο				Ο
Xerosis		Ο				Ο
Squamous cell carcinoma		Ο				Ο
Basal cell carcinoma		Ο				Ο
Malignant melanoma		Ο				Ο

*COSMIN* COnsensus-based Standards for the selection of health Measurement INstruments, *SAS* Skin Aging Score, *SCINEXA* SCore of INtrinsic and EXtrinsic skin Aging, *MAS* Merz Aesthetics Scale, *N/A* not applicable

#### Statistical analysis

The Pearson Chi-square and Student t tests were used to analyze categorical and continuous variables, respectively, where appropriate. Exploratory factor analysis (EFA) was performed to analyze the responses to explore the essential signs of skin aging based on the knowledge and experience of Thai dermatologists. EFA was chosen because the aim of this process was to identify linear group combinations of items that identify the optimal number of factors. The Kaiser-Guttman criterion [22] and scree test [23] were used to explore the optimal number of factors, which were used as an input for repeated EFA. Then, the factors were rotated to spread variability more evenly, so that all solutions were relatively the same. Items with high uniqueness were removed, whereas the remaining items had high factor loading, which should be at least 0.70, as this corresponds to approximately half of the variance of the variable being explained by the factor of interest. The retained items were grouped into factors, each of which was named based on the member items. Orthogonal (varimax) and oblique (promax) rotation provided similar results, but we decided to proceed with the latter because of the potential non-independent nature of the factors. An initial reliability test and item-based statistical analysis also were performed in conjunction with EFA [24]. Stata/SE version 15 (StataCorp) was used for all statistical calculations. p values < 0.05 were considered statistically significant.

### Results

Of 213 randomly selected dermatologists, 145 responded to the survey (response rate, 68.1%). Seventy-five percent of respondents were women, and their mean age was 35.2 years. Most respondents worked at a private clinic (46.2%), followed by a medical school (31.7%), public hospital (29.7%), and private hospital (22.8%). Mean work experience was 7.7 years, and they attended approximately 3 international conferences annually on average.

Both the Kaiser–Guttman criterion and scree plot suggested that 3 factors were optimal in our EFA (Table 3). Factor 1 comprised 8 signs of atrophy (wrinkles-superficial, wrinkles-deep, wrinkles-crisscross, reduced fat tissue, nasolabial folds, eye bags, lax appearance, and solar elastosis), which reflected dermis and soft tissue lesions. Factor 2 comprised 7 signs of discoloration (freckles, lentigines, melasma, venous lakes, purpura, telangiectasias, and milia). Factor 3 comprised of 3 malignant lesions.

**Table 3:** Factors related to skin aging

	Loading
Factor 1—atrophy	
Wrinkles-superficial	0.8587
Wrinkles-deep	0.8999
Wrinkles-crisscross	0.7437
Reduced fat tissue	0.8359
Nasolabial folds	0.7898
Eye bags	0.7696
Lax appearance	0.8027
Solar elastosis	0.7991
Factor 2—discoloration	
Freckles	0.8519
Lentigines	0.7946
Melasma	0.7565
Venous lakes	0.7056
Purpura	0.7346
Telangiectasias	0.7749
Milia	0.8056
Factor 3—malignancy	
Squamous cell carcinoma	0.8555
Basal cell carcinoma	0.8620
Malignant melanoma	0.8120

One-fifth (21.6%) of respondents were familiar with the SAS, compared with 11.2% and 9.4% who were familiar with MAS and SCINEXA, respectively. Average Likert scores were 2.54, 2.26, and 2.24 for the SAS, MAS, and SCINEXA, respectively. SAS was significantly better known than MAS (p < 0.001) and SCINEXA (p < 0.001); there was no difference between MAS and SCINEXA (p = 0.59).

### Discussion

Although more than 100 skin aging assessment scales have been introduced, none has gained sufficient popularity to become widely used. This is supported by our finding that a minimal number of Thai dermatologists were familiar with even the methodologically robust scales. Many reasons for this finding are possible. First, the terminology may be unclear and the assessment results may be complicated, requiring competent assessors with special training. For example, the term inability to redden [13], pigment spot [15], and benign tumor [12] could be interpreted in many different ways, making the scale unreliable. Second, some scales may be too comprehensive for real-life clinical practice. For instance, SCINEXA covers almost all signs of aging, and of the 13 studies that used this scale, only two completed the full assessment [25, 26]. Potential reasons were its complexity and dependency on competent assessors with special training [12]. Additionally, some scales required permission before use, whereas other scales leave out key skin aging signs, such as pigmentation and benign tumors.

We propose a simplified Global Subjective Skin Aging Assessment (GS2A2) score comprising 3 factors, which are based on empirical evidence from dermatologists in practice and representative of the pathophysiologic changes that occur during the skin aging process. The term “atrophy” comprises reduction of elastic fibers as well as changes in collagen components and subcutaneous tissues (fat, muscle, and bone), leading to skin signs of wrinkles, solar elastosis, reduced fat tissue, nasolabial folds, etc. The term “discoloration” includes melanin, changes in vascular pigmentation, and keratin-filled cysts. All common skin aging malignancies are included in factor 3. Based on these 3 factors, skin aging is multidimensional, and one skin sign is insufficient to express the skin aging process in general.

The GS2A2 score includes factors relevant to skin aging from the perspective of dermatologists, and it is easily calculated as a 3-factor score. Although each item of the score is assessed independently, the final summative number is used for representing the overall skin aging of a person as perceived by the dermatologist. This approach mimics a real-life situation, in which a layperson would justify the skin aging condition of another person from overall appearance; rather than from area-by-area assessment usually performed in a research study. Given a 5-point Likert scale for each item (from 1-Strongly disagree to 5-Strongly agree), the total score for this 18-item scale may range from 18 to 90. While the change of the total score can be a simple quantification of skin aging improvement of a subject, the score of each factor could help identify specific needs for a further correction in order to look younger. Nonetheless, as this is still an initial phase of scale development, the score should be further tested for validity and reliability before it can be used by a dermatologist to perform a visual assessment of overall skin aging condition of a subject in a simple summative number. The summary score may be used for assessing a cosmetic product that claims to have a global anti-aging effect, whereas a factor-based score could be used to differentiate whether the change in overall clinical outcomes was because of atrophy, discoloration, or malignancy. Moreover, standard terminology is used for each factor, resulting in better inter-rater reliability, which should be proven by further study. These results would support the GS2A2 score as a simple and informative tool. This tool is particularly relevant for Asian skin. However, it should be applied to non-Asian skin and/or non-Thai dermatologists. This score can also be used to measure overall anti-aging effects before and after dermatologic treatment, cosmetic products, or non-surgical skin rejuvenating procedures, such as laser therapy or microdermabrasion that claim to minimize overall skin aging. For example, the clinical effects of a multi-component nutritional supplement for photo-aged skin could be evaluated using the GS2A2 score instead of the Glogau classification system, which reflects only anti-wrinkle effects and not overall anti-aging effects. However, as the subjectivity of this score could be affected by many factors, the GS2A2 score should be used as a before-and-after comparison rather than as an average across individuals. Because this score was developed based on inputs from a representative group of Thai dermatologists who have experience with Asian patients, GS2A2 score is particularly relevant for Asians.

### Conclusions

The GS2A2 score was empirically generated based on multiple signs of skin aging and the perceptions of certified dermatologists. It is a simple numerical score that can be used to evaluate the anti-aging effects of a cosmetic product or dermatologic intervention. This score can be helpful for dermatologists to evaluate skin aging in clinical practice and research to compare treatment outcomes. It should be tested further for validity and reliability.

## Limitations

This study presented only the initial phase of scale development. As suggested by COSMIN, the GS2A2 score should be tested further for validity and reliability. Moreover, it should be conducted in other ethnicities to improve its generalizability.

## Additional file

**Additional file 1.** Global Subjective Skin Aging Assessment (GS2A2).

## Data Availability

All data generated or analysed during this study are included in this published article.

## Abbreviations

COSMIN: COnsensus-based Standards for the selection of health Measurement INstruments
EFA: exploratory factor analysis
GS2A2: Global Subjective Skin Aging Assessment
MAS: Merz Aesthetics Scale
SCINEXA: SCore of INtrinsic and EXtrinsic skin Aging
SAS: Skin Aging Score
