# Supplementary material for: Development of a Global Subjective Skin Aging Assessment score from the perspective of dermatologists
Source: BMC Res Notes. 2019 Jun 28;12:364. doi: 10.1186/s13104-019-4404-z (PMC6599371; doi:10.1186/s13104-019-4404-z)
Supplement: Supplementary file 1 — Additional file 1. Global Subjective Skin Aging Assessment (GS2A2). [file 13104_2019_4404_MOESM1_ESM.docx]

Thai dermatologist survey of skin aging assessment

1. Demographic Characteristics, Clinical Training & Experience

Age … years

Gender O Male O Female

Province ……

Education [ ] Board Certifications

[ ] Dermatology Resident in Training

[ ] Doctor of Philosophy in Dermatology

[ ] Master of Science in Dermatology

[ ] Dermatology Fellow (2-year Program)

[ ] Dermatology Fellow (1-year Program)

[ ] Diploma in Dermatology

[ ] General Practitioner

Work Experience … years

Work Setting [ ] Medical School [ ] Public Hospital

[ ] Private Hospital [ ] Private Clinic

2. I think that each of the following sign and disease are essential for skin aging diagnosis

|  | Strongly  Disagree | Disagree | … | Agree | Strongly  Agree |
| --- | --- | --- | --- | --- | --- |
| Wrinkle – Superficial |  |  |  |  |  |
| Wrinkle – Deep |  |  |  |  |  |
| Wrinkle – Criscross |  |  |  |  |  |
| Reduced Fat Tissue |  |  |  |  |  |
| Nasolabial Folds |  |  |  |  |  |
| Eye Bag |  |  |  |  |  |
| Ptosis of Upper Eye Lids |  |  |  |  |  |
| Yellowish Discoloration |  |  |  |  |  |
| Lax Appearance |  |  |  |  |  |
| Solar Elastosis |  |  |  |  |  |
| White Linear (Pseudoscar) |  |  |  |  |  |
| Cutis Rhomboidalis Nuchae |  |  |  |  |  |
| Freckles |  |  |  |  |  |
| Lentigines |  |  |  |  |  |
| Melasma |  |  |  |  |  |
| Uneven Pigment |  |  |  |  |  |
| Guttate Hypermelanosis |  |  |  |  |  |
| Venous Lakes |  |  |  |  |  |
| Purpura |  |  |  |  |  |
| Telangiectasias |  |  |  |  |  |
| Milia |  |  |  |  |  |
| Sebaceous Hyperplasia |  |  |  |  |  |
| Senile Comedones |  |  |  |  |  |
| Favre-Racouchot Syndrome |  |  |  |  |  |
| Actinic Keratosis |  |  |  |  |  |
| Squamous Cell Carcinoma |  |  |  |  |  |
| Basal Cell Carcinoma |  |  |  |  |  |
| Malignant Melanoma |  |  |  |  |  |
| Xerosis (Dry Skin) |  |  |  |  |  |

3. I am familiar with the following scales

[ ] Skin Aging Score

[ ] Merz Aesthetics Scale

[ ] SCore of INtrinsic and EXtrinsic Skin Aging
